# Long-lasting residual efficacy of a new indoor residual spraying product, VECTRON^™^ T500 (broflanilide), against pyrethroid-resistant malaria vectors and its acceptance in a community trial in Burkina Faso

**DOI:** 10.1186/s13071-024-06577-y

**Published:** 2024-11-23

**Authors:** Aristide Sawdetuo Hien, Koama Bayili, Samina Maiga, Welbeck Oumbouke, Jean Birba, Dieudonné Diloma Soma, Adissa Ya Ouattara, Delphine Ouissamien Karama, Marlize Coleman, Janneke Snetselaar, Graham Small, Shinya Niimi, Kawase Ayumi, Sidzabda Kompaoré, Katsutoshi Tsuchiya, Roch Kounbobr Dabiré, Abdoulaye Diabaté

**Affiliations:** 1 Institut de Recherches en Sciences de la Santé, Centre National de la Recherche Scientifique et Technologique, Ouagadougou, Burkina Faso; 2 Innovative Vector Control Consortium (IVCC), Liverpool, UK; 3 Mitsui Chemicals Crop & Life Solutions, Inc., Tokyo, Japan; 4 Permanent Secretariat for Malaria Elimination, Ouagadougou, Burkina Faso

**Keywords:** VECTRON^™^ T500, Actellic^®^ 300CS, Indoor residual spraying, *Anopheles gambiae* s.l., Efficacy, Burkina Faso

## Abstract

**Background:**

The WHO Global Malaria Programme advocates for a comprehensive, strategic approach to managing insecticide resistance, highlighting the importance of using multiple insecticides with different modes of action through rotations and combinations. To slow the spread of resistance, it is essential to develop and evaluate new formulations that feature unique modes of action and extended residual effects. Addressing this need, Mitsui Chemicals Crop & Life Solutions, Inc., developed VECTRON™ T500, a new indoor residual spraying (IRS) formulation using broflanilide, applied at a dosage of 100 mg AI/m^2^. This formulation was tested in a Phase III community trial, alongside Actellic^®^ 300CS, a commonly used IRS product containing pirimiphos-methyl, applied at the recommended dosage of 1000 mg AI/m^2^.

**Methods:**

Monthly WHO wall cone bioassays were performed to assess the efficacy of the interventions using three mosquito strains: the laboratory-bred, insecticide-susceptible *Anopheles gambiae *s.s. Kisumu strain, the insecticide-resistant *Anopheles coluzzii* VKPer strain, and wild *Anopheles gambiae* s.l. mosquitoes from the Vallée du Kou, where the study was conducted. Vector surveillance was carried out to compare the results between sites treated with VECTRON™ T500, Actellic^®^ 300CS, and an untreated control site. In addition, any reported adverse effects were closely monitored to evaluate the community’s acceptance of VECTRON^™^ T500.

**Results:**

VECTRON™ T500 consistently achieved 100% mortality across all wall types for both susceptible and resistant mosquito strains over the 12-month period. In comparison, Actellic^®^ 300CS induced < 80% mortality for both strains, irrespective of the wall substrate. When assessing delayed mortality in *An. gambiae* s.l. mosquitoes collected from sites treated with Actellic^®^ 300CS (VK1) and VECTRON™ T500 (VK3), a statistically significant difference was noted after a 72-h holding period compared to the control site (RR = 0.51, CI95% = [0.31–0.6], *P* = 0.0026). Additionally, no adverse events were reported in households sprayed with VECTRON™ T500.

**Conclusions:**

The residual efficacy of VECTRON^™^ T500 extended for 12 months post-spraying, effectively covering the full malaria transmission season while maintaining high mortality rates in pyrethroid-resistant malaria vectors. VECTRON^™^ T500 demonstrated non-inferiority in performance compared to Actellic^®^ 300CS, the standard reference product. This new IRS formulation has the potential to play a crucial role in managing insecticide resistance by being integrated into a rotational strategy alongside other IRS products containing insecticides with different modes of action.

**Graphical Abstract:**

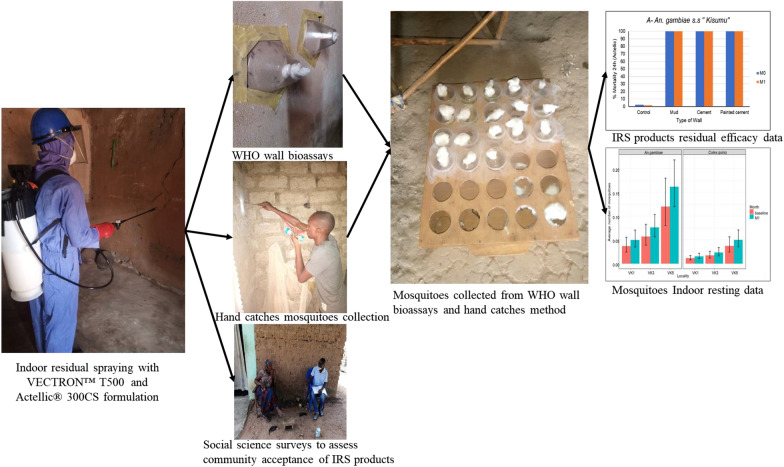

## Background

The effectiveness of indoor residual spraying (IRS) as a malaria control strategy is increasingly compromised by the growing insecticide resistance among malaria vectors, primarily due to the limited number of insecticides approved for public health use [1]. Pyrethroid resistance has become widespread across Africa, significantly undermining the reliability of this insecticide class for IRS. Consequently, the World Health Organization (WHO) has recommended against using pyrethroid-based IRS in regions with high coverage of long-lasting insecticide-treated nets (LLINs) [2]. Moreover, resistance has rapidly escalated among alternative insecticide classes, such as carbamates and organophosphates, which were previously the only viable options for IRS [3–5]. To combat the escalating threat of insecticide resistance, malaria control programs are encouraged to adopt a strategy that involves rotating insecticides with different modes of action [2]. This rotation reduces the selection pressure on any single insecticide, allowing resistance to decline over time due to the fitness costs associated with resistance mechanisms when the selection pressure is removed. The successful implementation of this strategy necessitates the development of new classes of insecticides to support malaria elimination efforts over the next 2 decades [6, 7].

Historically, most IRS operations in Africa relied on organophosphate or neonicotinoid-based products. Actellic^®^ 300CS, an organophosphate formulation of pirimiphos-methyl, received WHO's recommendation in 2013 [8, 9]. Additionally, several neonicotinoid-based IRS formulations, including clothianidin (SumiShield^®^ 50WG, Klypson 500WG) and combinations with deltamethrin (Fludora^®^ Fusion, 2GARD), have been listed by the WHO Prequalification Unit Vector Control Product Assessment Team (WHO PQT/VCP) [10]. A promising new IRS product, VECTRON™ T500, has been developed by Mitsui Chemicals Crop & Life Solutions, Inc. (MCCLS), and contains broflanilide, a meta-diamide insecticide [11]. Broflanilide has shown significant potential in controlling vector populations, particularly those resistant to pyrethroids and other insecticide classes [12]. It operates by targeting the GABA receptor in the nervous systems of mosquitoes and is classified under Group 30: GABA-gated chloride channel allosteric modulators by the Insecticide Resistance Action Committee (IRAC) [13]. The development of VECTRON™ T500 was supported by the Innovative Vector Control Consortium (IVCC) and involved collaboration with institutions such as the London School of Hygiene and Tropical Medicine (LSHTM), Kilimanjaro Christian Medical University College (KCMUCo), and the Centre de Recherche Entomologique de Cotonou (CREC) [14]. Laboratory and semi-field studies conducted in Benin, Burkina Faso, and Tanzania have confirmed the efficacy of VECTRON^™^ T500 against pyrethroid-resistant Anopheles species [15–17]. Consequently, it was added to the WHO's list of prequalified IRS products [18], making it available for malaria control programs. Importantly, no cross-resistance to broflanilide has been detected among African malaria vectors, underscoring its suitability for large-scale deployment [15, 19, 20]. Recently, a community-based trial was conducted to evaluate the large-scale deployment of VECTRON^™^ T500 in endemic regions [21].

This current trial aimed to provide new evidence supporting the product’s use, as recommended by WHO PQT/VCP, which mandates that national authorities and procurement agencies ensure IRS products meet WHO standards. In Burkina Faso, as well as in other countries under the Comité Permanent Inter-Etats de Lutte contre la Sécheresse dans le Sahel (CILSS), the Sahelian Pesticide Committee regulates pesticides, authorizing the import and use of insecticides for public health based on rigorous evidence. VECTRON™ T500 was tested in Burkina Faso for the first time to meet the requirements of this committee. The study sought to generate further evidence of the efficacy of VECTRON™ T500 against pyrethroid-resistant malaria vectors, evaluating its performance when applied to household walls compared to Actellic^®^ 300CS (pirimiphos-methyl), the reference IRS product. The trial emphasized assessing the residual efficacy of VECTRON^™^ T500 and its acceptance by the community as an effective malaria vector control tool.

## Methods

### Study sites and design

**Fig. 1:**
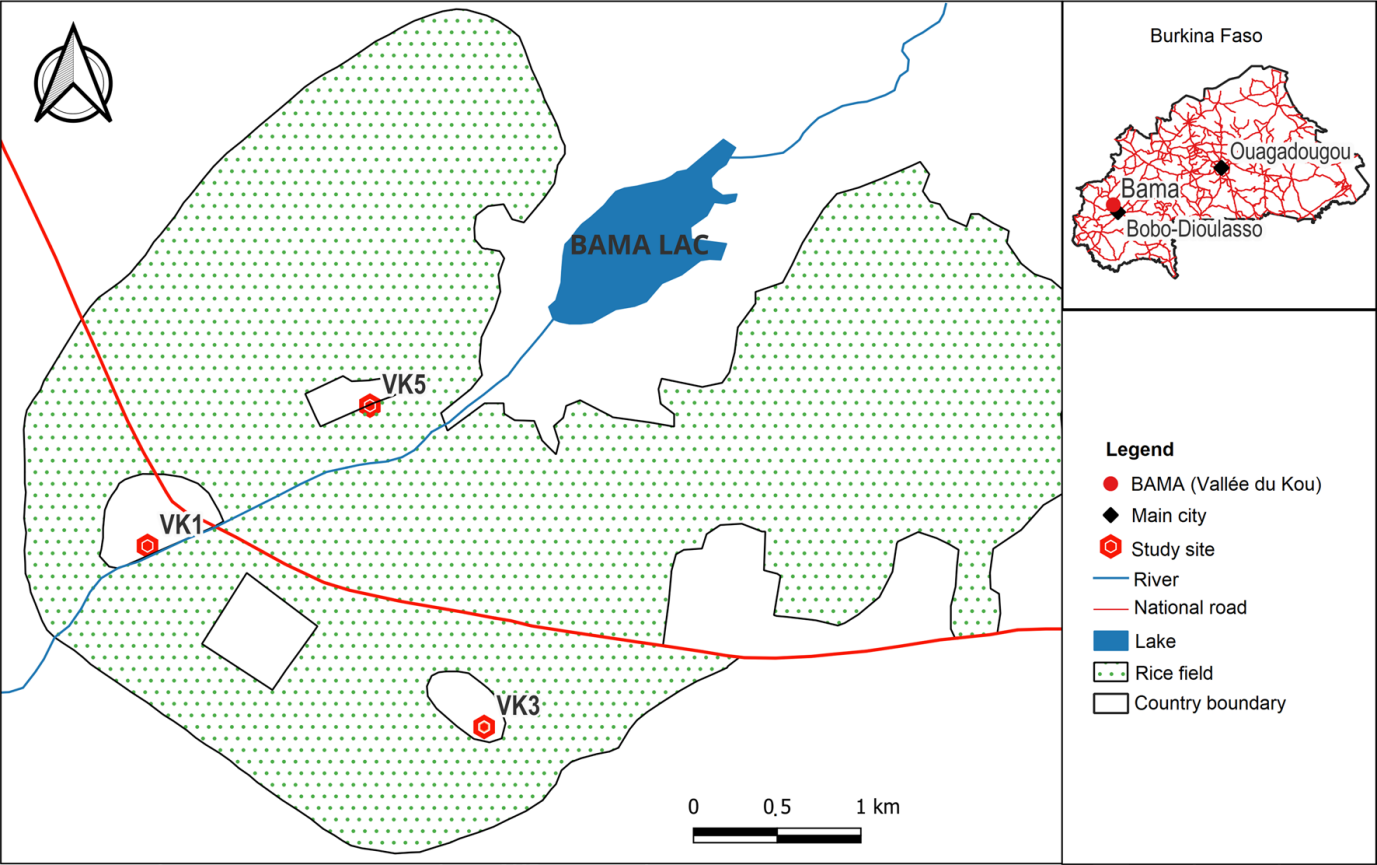
Study area and allocation of treatment

The study was carried out in the Vallée du Kou, located in the commune of Bama, southwestern Burkina Faso, about 30 km north of Bobo-Dioulasso. Three quarters within this rice-growing area were selected: VK5 (11°24’N; 4°23’W), VK3 (11°23′14″N; 4°24′42″W), and VK1 (11°24'N; 4°24′59"W) (Fig. 1). The residential structures in these areas consist of walls made from mud, concrete, or painted cement. With a population of 22,244 in 2020, Vallée du Kou lies within the Sudanian zone, which experiences a dry season from November to April and a rainy season from May to October, receiving an annual average rainfall of 1200 mm [22]. The region is a major rice-producing area with two cropping cycles each year. Rice plains are irrigated from January to May and June to November. While insecticide use is minimal in rice cultivation, nearby cotton fields rely heavily on insecticides for pest control. The irrigation system supports year-round water availability, creating permanent, highly productive mosquito breeding sites in the rice paddies. Additional breeding sites include natural depressions and reservoirs, which become active during the rainy season. Malaria transmission is perennial, peaking during the rainy season, with *Anopheles coluzzii* (formerly M-form of the *Anopheles gambiae* complex) being the dominant vector, coexisting with *An. gambiae* s.s. [23]. These local malaria vectors exhibit high resistance to pyrethroids and DDT due to the prevalent L1014F *kdr-*mutation (0.8–0.9) [24, 25]. Additionally, the ace-1R mutation is found in both species, with a higher frequency in *An. gambiae* s.s. (0.4) than in *An. coluzzii* (0.03) [26], indicating a multi-resistance profile in the local mosquito populations [27].

The study employed a cross-sectional observational design in selected rice-growing sites. Prior to the trial in April 2021, a census was conducted to identify eligible structures. Stratified randomization, based on wall type (mud, concrete, and painted cement), was used to select 30 houses in each site for baseline data collection, ensuring comparability across the trial arms. Of these, 15 houses per site (5 mud, 5 concrete, and 5 painted cement) were randomly chosen for initial data collection. Baseline analyses confirmed that the sites were similar in terms of vector density and wall types. After baseline data collection, indoor residual spraying (IRS) formulations were applied. VECTRON™ T500, a broflanilide-based product, was sprayed in VK3 at a dosage of 100 mg AI/m^2^, while the reference product, Actellic^®^ 300CS (pirimiphos-methyl), was applied in VK1 at 1000 mg AI/m^2^. VK5 served as the control site, where no IRS was conducted, though other standard vector control tools were in use. Actellic^®^ 300CS was selected as the reference product because of its widespread use in the national malaria control program [28] and its familiarity among the local population, unlike other IRS products such as SumiShield^®^ 50 WG [29] and Fludora^®^ Fusion WP-SB [30]. Monthly entomological surveys were conducted for 6 months (June to November 2021) corresponding to the malaria transmission season in 15 randomly selected houses (5 per wall type) in each site. Additionally, WHO wall cone bioassays were carried out in six houses per site (2 mud, 2 concrete, and 2 painted cement) to assess insecticide residual efficacy. Otherwise, a structured questionnaire was administered to 30 household heads in the sprayed sites to document any adverse effects and to assess community acceptance of VECTRON™ T500. These bioassays and community surveys continued for 12 months, from May 2021 to May 2022, providing valuable insights into the residual efficacy of the IRS intervention and community perception. In addition, given that the wall types differ from those documented in other studies performed that were hut experimental studies [15–19], this research also aimed to evaluate the residual efficacy of the insecticides used in real conditions for a prolonged period (12 months). Understanding this residual efficiency is crucial for establishing re-treatment cycles. A prolonged residual efficacy of the insecticides applied to these walls would be advantageous for the national malaria control program as it would not only be more cost-effective but also more suitable for implementation.

### Entomological surveys

During the post-spraying phase of the study, entomological data collection began 1 week after spraying and continued weekly until the final week of November 2021. Indoor resting mosquitoes were collected early in the morning (06:00–07:30 a.m.) using mouth aspirators and flashlights in 15 selected human dwellings per site (both sprayed and control). This method, considered one of the most effective for assessing indoor resting mosquito species, allowed for accurate estimates of mosquito density in anthropophilic (human-preference) areas. In each house, two well-trained mosquito collectors, assisted by a local guide from the study area, conducted the collection for 15–20 min. Mosquitoes were collected from walls, the undersides of beds, and every corner of the house. Consistency was maintained by using the same houses throughout the study, and mosquito collections were performed simultaneously across the three study sites. Dead mosquitoes were recorded immediately, while live mosquitoes were aspirated into cups and fed a 10% sugar solution. Each cup was labeled with detailed information such as location, wall type, and collection time. Delayed mosquito mortality was recorded at 24-, 48-, and 72-h post-collection, and mortality rates from sprayed sites were compared to those from the control site. The mosquitoes collected were counted and identified morphologically to species level using taxonomic keys [31]. Further analyses were conducted for blood meal source identification using direct enzyme-linked immunosorbent assays (ELISA) [32], which tested for human, bovine, pig, donkey, and sheep blood. Sibling species identification was achieved using the SINE PCR technique [33].

### Residual activity of IRS insecticides on house walls

WHO wall cone bioassays (WHO, 2006) were conducted to assess the efficacy of the IRS treatments at various time points: initially at T0 (1 week post-spraying in May 2021) for quality assurance, and then monthly from June 2021 (T1) to May 2022 (T12), to monitor residual efficacy. Three mosquito strains were used in these bioassays: the *Anopheles gambiae* s.s. Kisumu strain (susceptible), the *An. coluzzii* VKPer strain (resistant), and a wild population of *An. gambiae* s.l. collected from the study area. These wild mosquitoes were collected as larvae and reared to adulthood for testing. Due to limited availability of wild mosquitoes during the dry season (December onwards), the resistant *An. coluzzii* VKPer strain, which originated from Vallée du Kou and exhibited resistance to permethrin, was used from December 2021 to May 2022. In each test, two cones were affixed to the walls at different heights: 0.5 m (low), 1.0 m (middle), and 1.5 m (upper). These cones were randomly designated for either the susceptible or resistant mosquito strains. The bioassays were conducted in six houses at each site: two with mud walls, two with concrete walls, and two with painted cement walls. These same houses were used consistently throughout the study. For each bioassay, ten unfed female mosquitoes (2–5 days old) were aspirated into the cones and exposed to either treated walls (sprayed with VECTRON^™^ T500 or Actellic® 300CS) or untreated control walls for 30 min. Each month, at least 30 mosquitoes per strain were tested per site. Following exposure, the mosquitoes were placed into paper cups and fed a 10% sugar solution. Knockdown rates were recorded 60 min after exposure, and the mosquitoes were subsequently held in a controlled laboratory environment at 27 °C ± 2 °C and 80% ± 10% relative humidity. Mortality rates were recorded at 24, 48, and 72 h for mosquitoes exposed to VECTRON™ T500-treated walls and at 24 h for those exposed to Actellic^®^ 300CS-treated walls. Additionally, control cones containing ten mosquitoes were placed outside each sprayed house, positioned on a piece of each type of untreated wall in a shaded area, to ensure that the environmental conditions were consistent across all treatments.

### Insecticide susceptibility monitoring

Mosquitoes for insecticide susceptibility bioassays were sampled as larvae from breeding sites within the study area and reared to the F1 generation in the insectary before testing. The susceptibility of the vector population to relevant insecticides was assessed between August and October 2021, during which there was a high abundance of mosquito larvae in the breeding sites. Three pyrethroid insecticides were tested at their diagnostic concentrations: permethrin (0.75%), deltamethrin (0.05%), and alpha-cypermethrin (0.05%). Additionally, the intensity of pyrethroid resistance was monitored using treated papers of alpha-cypermethrin, deltamethrin, and permethrin at 5 and 10 times the diagnostic concentration in WHO tubes.

Prior to the field susceptibility assays, quality control was performed by randomly selecting two impregnated papers from each box and testing them against laboratory-maintained susceptible *An. gambiae* s.s. Kisumu strain mosquitoes. The susceptibility of field-sampled mosquitoes to broflanilide was assessed using seven different doses: 0.781 µg, 1.563 µg, 3.125 µg, 6.25 µg, 12.5 µg, 25 µg, and 50 µg of broflanilide per bottle. The diagnostic dose of pirimiphos-methyl used in assays was 20 µg per bottle. CDC bottle bioassays were conducted using unfed female *An. gambiae* s.s. Kisumu and wild-type *An. gambiae* s.l. mosquitoes, collected in the larval stage from the study area. The stock solution for broflanilide was prepared by serially diluting the technical grade of broflanilide at a concentration of 100 mg in 200 ml of acetone, with the adjuvant Mero^®^ (Bayer CropScience) included at 800 ppm. One milliliter of each stock solution was used to coat 250-ml Wheaton bottles, with six bottles prepared for each dose, following the CDC bottle bioassay guidelines. For pirimiphos-methyl, a stock solution was prepared at 200 µg/ml by dissolving 20 mg of pirimiphos-methyl in 100 ml of acetone. This stock solution was then diluted (1 ml in 9 ml of acetone) to provide the working solution at the discriminating concentration of 20 µg per bottle. In the bottle bioassays, approximately 100–150 female mosquitoes per insecticide dose were exposed for 1 h in cohorts of 25 mosquitoes per bottle for broflanilide and for 30 min for pirimiphos-methyl, according to CDC guidelines. The mosquitoes were held in a controlled environment at 27 °C ± 2 °C and 80 ± 10% relative humidity. Mortality rates were recorded after 72 h for broflanilide, based on preliminary evidence of its slower mode of action, and after 24 h for pirimiphos-methyl.

### Assessment of perceived adverse events and acceptability

Community acceptability of the insecticide treatments was assessed through structured interviews with 30 heads of households selected from each study site. This approach ensured a comprehensive understanding of community perceptions and potential health impacts associated with using insecticides. Data collection occurred 1 week post-spraying and continued at monthly intervals until the end of the study in the sprayed areas. Alongside the evaluation of the insecticide's impact on target vector species, various factors that could influence acceptability were documented, including: (i) visible insecticide stains by observations regarding any noticeable stains left on walls after spraying; (ii) odor by smelling the presence of any unpleasant odors emanating from the insecticide; (ii) irritation symptoms by reporting any skin and nasal irritation among household members. Additionally, the impact of the sprayed insecticides on other household insects, including nuisance species, was recorded to understand the broader ecological effects of the treatments. To further assess the safety of the insecticides, a separate questionnaire was administered to insecticide handlers, including spray operators and supervisors. This questionnaire was conducted both before and after the spray applications in the treated areas to gauge any perceived side effects from handling the insecticides. Moreover, study participants were encouraged to contact the study team if they experienced any health issues they believed were related to the IRS applications.

### Data analysis

The raw data collected during the study were double-entered and managed using a predefined Excel database. All statistical analyses were performed using Stata version 16.0 (Stata Corp.; College Station, TX, USA).

#### In situ wall cone bioassays

Mortality data from the wall cone bioassays were pooled across households for each substrate type. The results were plotted against the number of months since spraying to evaluate the residual efficacy of each IRS treatment. Residual efficacy was defined as the number of months for which overall mortality for each mosquito species and wall substrate type remained ≥ 80% [34]. When control group mortality rates ranged between 5 and 20%, Abbott’s formula was applied to correct the results.

#### CDC bottle bioassays

The susceptibility threshold for mortality of malaria vectors was defined as 98–100% [35]. Mortality rates between 90 and 97% suggested resistance, warranting further investigation, while mortality < 90% indicated confirmed resistance [36].

#### Main bionomics parameters

The relative abundance of indoor resting vectors was determined and compared across wall substrate types and study sites. The number of female *Anopheles* mosquitoes was assessed based on the collection period (before or after IRS) and intervention status (sprayed or non-sprayed), treating sites as a random effect.

Indoor resting density (IRD) was calculated as the mean number of *An. gambiae* s.l. collected per house (total number of *An. gambiae* s.l. collected via hand catches divided by the total number of surveyed houses).

The immediate mortality was defined as the proportion of mosquitoes found dead at each weekly collection, with details recorded on location, wall type, and hour of collection. The delayed mortality was recorded up to 96 h and compared between sprayed and control sites. The holding days were represented by number of days from day 1 “D1” to day 4 “D4.”

Blood feeding rate was defined as the proportion of blood-fed female mosquitoes relative to the total number collected in the huts. Blood feeding inhibition due to insecticide was calculated using the formula: blood feeding inhibition = 100 × (*Pc* − *Pt*/*Pc*) where *Pc*​ is the percentage blood feeding in control houses and *Pt*​ is the percentage blood feeding in sprayed houses.

The Tukey’s method of analysis using relative ratio (RR) was used to compare quantitative data. Chi-square tests were used for comparing qualitative or binomial data between sites. A 5% significance level was set for all statistical testing.

## Results

### Mosquito species composition and density

**Fig. 2:**
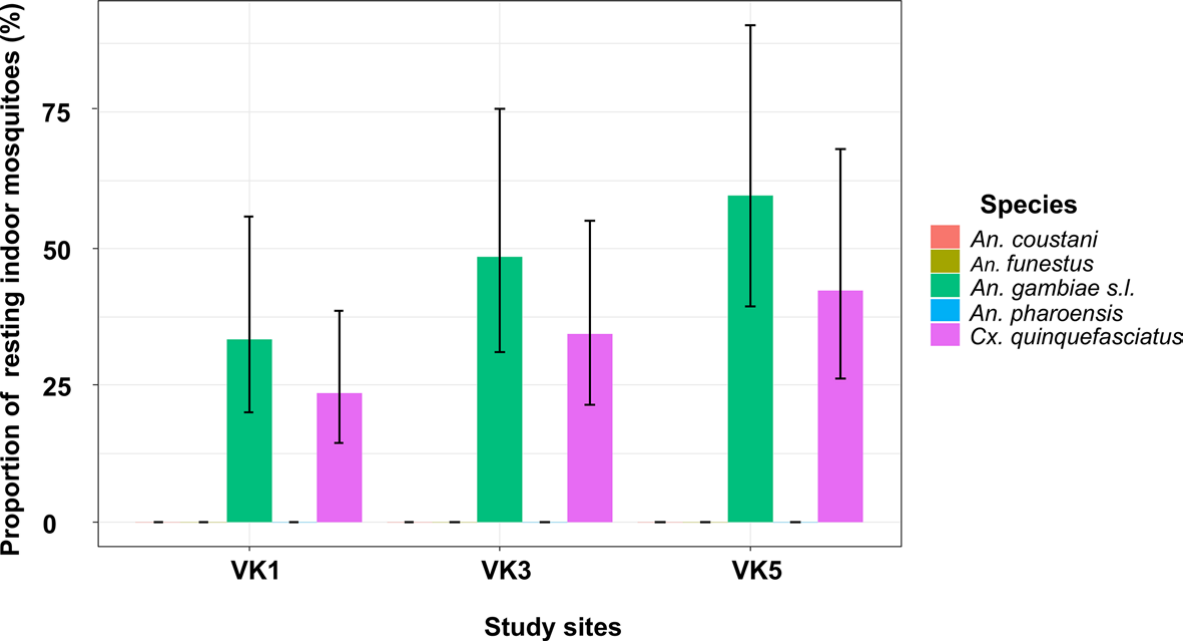
Vector species collected in VK1 and VK3 and VK5 during baseline collections (prior to IRS intervention)

Prior to spraying, the baseline collection (M0, corresponding to May 2021) revealed no significant difference in the mean number of *An. gambiae* s.l. caught in indoor collections (indoor resting density, IRD) across the three study sites: VK5 (IRD: 9.5 ± 3.1 *An. gambiae* s.l. per house/hour) versus VK1 (IRD: 5.9 ± 2.6 *An. gambiae* s.l./h) with a relative ratio (RR) of 1.79 (95% CI [0.88–3.61], *P* = 0.12); control (IRD: 9.5 ± 3.1 *An. gambiae* s.l./h) versus VK3 (IRD: 6.7 ± 2.5 *An. gambiae* s.l./h) (RR = 1.23, 95% CI [0.64–2.37], *P* = 0.73); and VK3 (IRD: 6.7 ± 2.5 *An. gambiae* s.l./h) versus VK1 (IRD: 5.9 ± 2.6 *An. gambiae* s.l./h) with RR = 0.68 (95% CI [0.33–1.40], *P* = 0.43) (Fig. 2). Consequently, the three sites exhibited similar trends in the density of indoor resting mosquitoes and were designated as intervention and control sites for subsequent surveys. Otherwise, the mean number of *Culex quinquefasciatus* was similar to that of *An. gambiae* s.l. collected during baseline data collection (*P* = 0.81).

Following the application of the IRS treatments (from June to November 2021), a total of 12,084 *Culicidae* mosquitoes were collected across all three study sites. The breakdown of these collections was as follows: 1935 from the Actellic^®^ 300CS site, 2327 from the VECTRON™ T500 site, and 7822 from the control site. The species composition of the Culicidae collected included 11,584 Anopheline and 500 Culicine mosquitoes. Among the Anopheline specimens, *Anopheles gambiae* s.l. was the predominant species, accounting for 96.46% (11,175/11,584) of the total, followed by *Anopheles pharoensis* (8/11,584), *An. coustani* (6/11,584), and *An. funestus* (4/11,584). PCR identification of *An. gambiae* s.l. collected from the three sites between June and November 2021 indicated that *An. coluzzii* was the only species identified (100%) throughout the survey period.

**Fig. 3:**
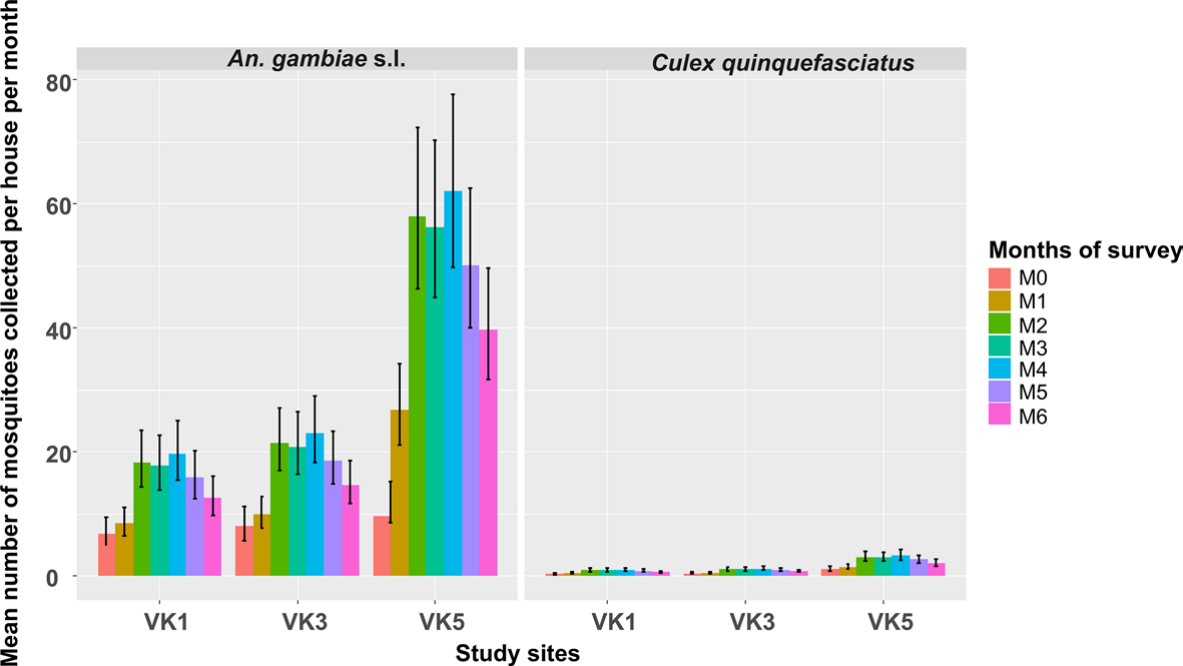
Mean number of mosquitoes collected per houses and per month according to treatment

The mean indoor resting densities (IRD) of *An. gambiae* s.l. varied according to treatment and the month of collection. The highest densities were recorded in VK5 (control site) during M4 (September), with 63 ± 10.1 *An. gambiae* s.l. per house per month (/h/m) (Fig. 3), while the mean IRD in the sprayed sites for the same month was slightly lower, with 24 ± 4.8 and 19 ± 3.6 *An. gambiae* s.l./h/m in VK1 and VK3, respectively. Moreover, the mean IRD significantly increased in the control site from June to November 2021 compared to the two sprayed sites (Actellic^®^ 300CS and VECTRON™ T500). The insecticide applied in the intervention sites resulted in a two-fold reduction in the IRD of *An. gambiae* s.l. compared to that observed in the control site (RR = 0.3, 95% CI [0.2–0.38], *P* = 0.0001). However, when comparing the mean IRD between the two sprayed sites (Actellic^®^ 300CS and VECTRON^™^ T500), the IRDs of *An. gambiae* s.l. were found to be similar (RR = 0.88, 95% CI [0.65–1.20], *P* = 0.63). In contrast, the indoor resting densities of *Cx. quinquefasciatus* did not exceed five mosquitoes per house per month.

### Impact of IRS on mortality rates of wild free‑flying pyrethroid‑resistant *An. gambiae s.l.* Vallée du Kou

The baseline data analysis indicated that the three sites exhibited comparable indoor resting densities of mosquitoes prior to the intervention. The recorded immediate mortality rates of mosquitoes were 38.89% (CI 95% 36.7–41.1) at VK1 (Actellic^®^ 300CS site), 75.4% (CI 95% 73.7–77.2) at VK3 (VECTRON^™^ T500 site), and 23.1% (CI 95% 22.1–24.1) at VK5 (control site) (Table 1). A statistically significant difference in mortality was observed between the treatment sites (VK1 and VK3) and the control site, with a relative risk (RR) of 0.51 (CI 95% [0.31–0.6], *P* = 0.0026).

**Table 1:** Immediate mortality of free-flying *Anopheles gambiae* s.l. collected from IRS-sprayed and control sites after spray applications

Treatment	VK1 (Actellic^®^ 300CS)			Overall	VK3 (VECTRON^™^ T500)			Overall	VK5 (Control)			Overall
Wall substrate type	Mud	Concrete	Painted cement		Mud	Concrete	Painted cement		Mud	Concrete	Painted cement	
No. collected	731	502	613	1846	778	778	727	2283	2395	2179	2472	7046
No. dead	260	240	218	718	581	589	552	1722	457	646	526	1629
% Mortality (CI95%)	35.6 (32.1–39)	47.8* (43.4–52.2)	35.6 (31.8–39.4)	38.9 (36.7–41.1)	74.7 (71.6–77.7)	75.7 (72.7–78.7)	75.9 (72.8–79)	75.4 (73.7–77.2)	19.11 (17.5–20.7)	29.6 (27.7–31.6)	21.3 (19.7–22.9)	23.1 (22.1–24.1)

Value with * means a statistically significant difference was observed compared to another group at the 5% level

**Fig. 4:**
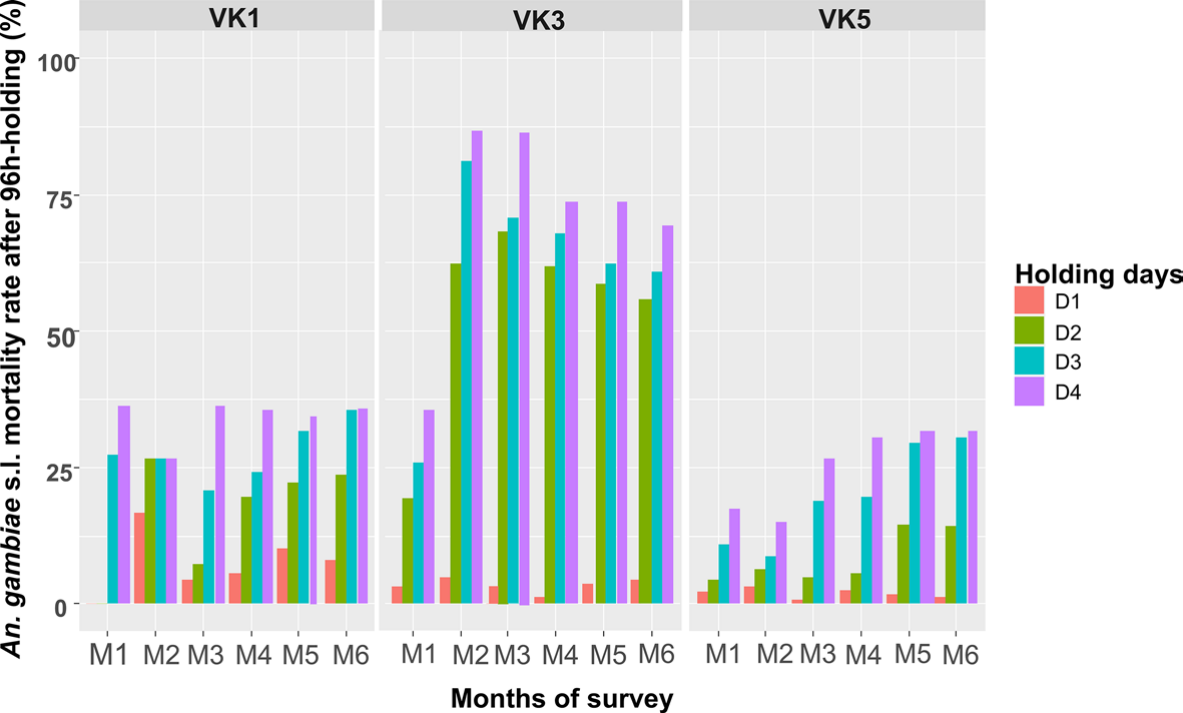
Average immediate and delayed *Anopheles gambiae* s.l. mortality rate in control (VK5) and sprayed sites (VK1: Actellic^®^300CS and VK3: VECTRON^™^ T500)

At baseline, the mortality rate of *An. gambiae* s.l. was low across all sites but exhibited a steady increase from the 1st to 7th month post-spraying with VECTRON^™^ T500 (Fig. 4). Notably, an increase in mortality was observed from 1 to 4 days of the holding period, suggesting a delayed mortality effect attributed to the slower mode of action of broflanilide, the active ingredient in the VECTRON^™^ T500 IRS product. Moreover, VECTRON^™^ T500 resulted in higher mortality rates among *An. gambiae* s.l. mosquitoes compared to Actellic^®^ 300CS, which demonstrated mortality rates similar to those observed at the control site.

### Impact of IRS on *An. gambiae s.l.* blood-fed success in Kou Valley

A total of 7198 female *An. gambiae* s.l. mosquitoes from the three sites were assessed for their blood-fed success over months post-spraying (Table 2). In terms of blood feeding, the control site yielded a higher number of blood-fed females (536) compared to both sprayed sites (73 in VK3 and 80 in VK1; *P* < 0.0001) (Table 2). The number of blood-fed females significantly decreased after the application of VECTRON™ T500 compared to the control site (χ^2^ = 236.03, df = 1, *P* < 0.0001). Specifically, the proportion of blood-fed female *An. gambiae* s.l. was nearly two-fold lower in the VECTRON^™^ T500 sprayed site (4.43% with CI 95% [4.1–8.7]) than in the control site (12.33% with CI 95% [11.3–17.6]) (Table 2). Consequently, the overall blood-feeding inhibition (BFI) rate for *An. gambiae* s.l. was calculated at 64.01% (CI 95% [57.1–72.2]) because of the IRS effect of VECTRON^™^ T500, while the BFI rate attributable to Actellic^®^ 300CS was 34.14% (CI 95% [30.6–38.7]) (Table 2). Thus, VECTRON™ T500 demonstrated a significantly higher BFI compared to Actellic^®^ 300CS.

**Table 2:** *Anopheles gambiae* s.l. female blood-feeding rates and blood-feeding inhibition (BFI) in sprayed sites

Treatment	Total females examined	Total blood-fed females	% Blood-fed females (CI 95%)	BFI (CI 95%)
Unsprayed site (VK 5)	4567	536	11.7 (10.8–12.7)	–
VECTRON™ T500 sprayed site (VK3)	1646	73	4.4 (3.4–5.4)	64.01* (57.1–72.2)
Actellic^®^ 300CS sprayed site (VK1)	985	80	8.1 (6.4–9.8)	34.14 (30.6–38.7)
Total	7198	689		

Value with * means a statistically significant difference was observed compared to another group at the 5% level

### Residual efficacy of IRS products against laboratory susceptible (*An. gambiae *s.s. *Kisumu*) and resistant strains (*An. coluzzii VKPer*) and wild *An. gambiae s.l.* population in WHO wall cone bioassay

The mortality rate in mosquitoes exposed to the control wall during cone bioassays consistently remained < 5% across all testing rounds.

**Fig. 5:**
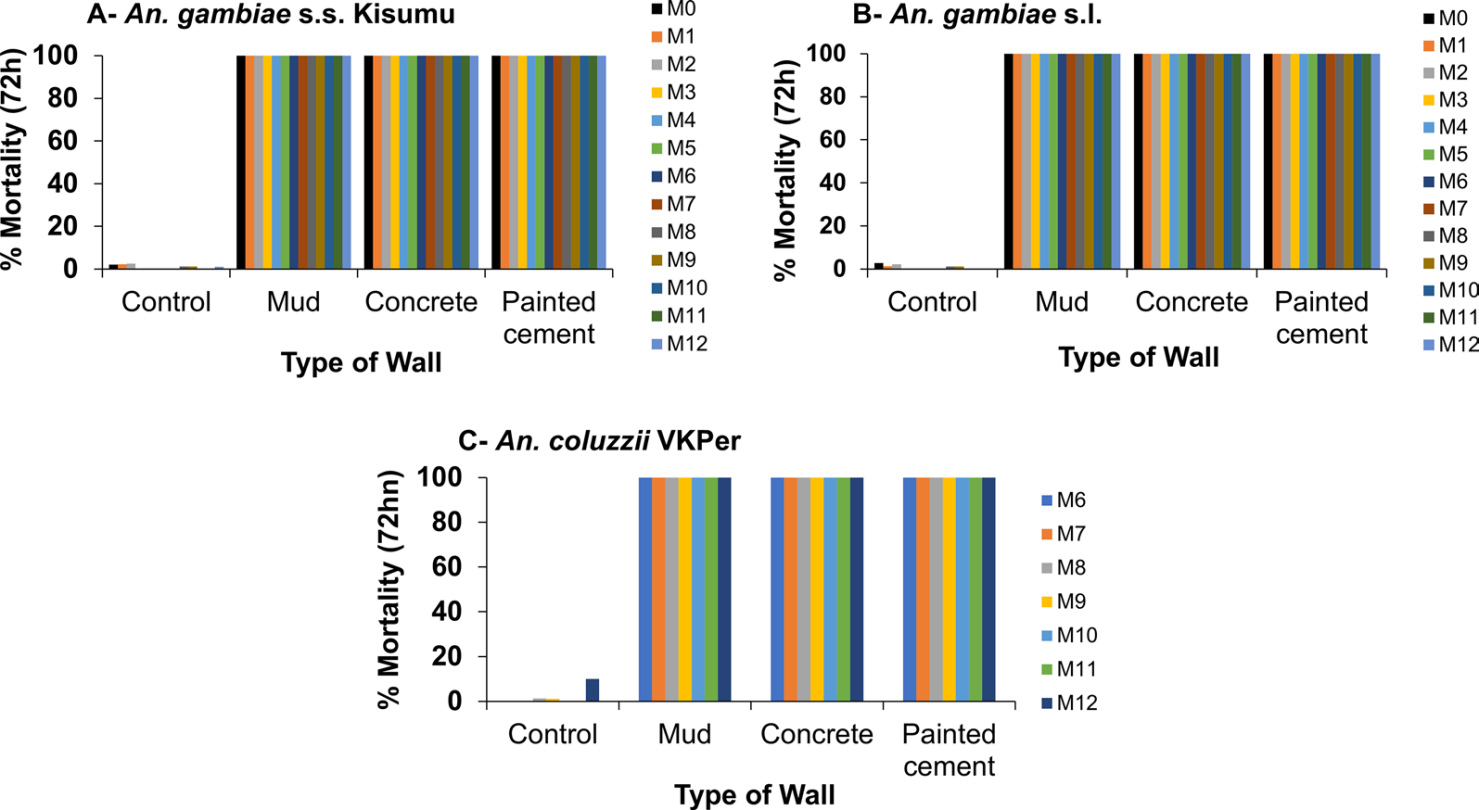
72-h mortality of susceptible *Anopheles gambiae* s.s. Kisumu strain (**A**), *An. gambiae* s.l. Vallée du Kou strain (**B**), and pyrethroid-resistant *An. coluzzii* VKPer strain (**C**) exposed to VECTRON™ T500 in sprayed mud, concrete, and painted cement walls from May 2021 to May 2022

The residual efficacy of VECTRON^™^ T500 was notably high throughout the 12 months following spraying, achieving 100% mortality at 72 h post-exposure in cone assays for all three mosquito strains susceptible (*An. gambiae* s.s. Kisumu), resistant (*An. coluzzii* VKPer), and wild (*An. gambiae* s.l.) across all wall substrates (mud, concrete, and painted cement) in VK3 (Fig. 5).

**Fig. 6:**
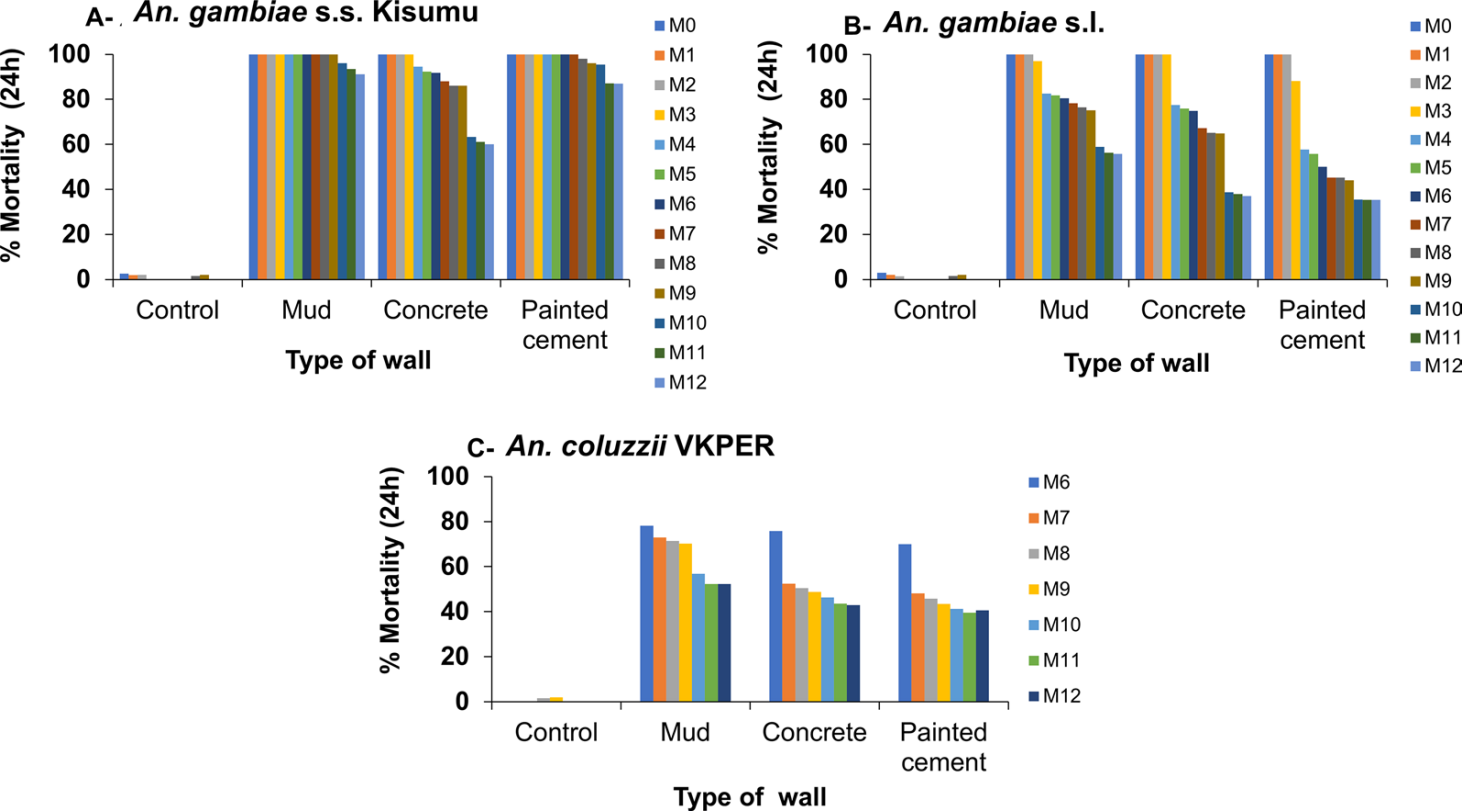
24-h mortality of susceptible *Anopheles gambiae* s.s. Kisumu strain (**A**), *An. gambiae* s.l. Vallée du Kou strain (**B**), and pyrethroid-resistant *An. coluzzii* VKPer strain (**C**) exposed to Actellic® 300CS in sprayed mud, concrete, and painted cement walls from May 2021 to May 2022

The reference product, Actellic^®^ 300CS, was also evaluated on these wall types using the same mosquito strains. Mortality rates for Actellic^®^ 300CS began to decline from the 9th to 12th month post-spraying, with recorded mortality of 92% on mud surfaces (Fig. 6). For the susceptible Kisumu strain on painted cement walls treated with Actellic^®^ 300CS, mortality decreased after the 7th month (94% mortality), while on concrete walls, it declined after the 3rd month (96%), with variations depending on the wall type. At the 12th month post-spraying, the lowest mortality rate was observed on concrete walls, where only 60% mortality was recorded for *An. gambiae* s.s. Kisumu and < 40% for the wild *An. gambiae* s.l. Vallée du Kou (Fig. 6). Additionally, the resistant *An. coluzzii* VKPer strain was assessed on Actellic^®^ 300CS treated walls up to 6 months post-spraying, exhibiting a similar trend to that of the wild strain, with mortality rates remaining < 80% from 6 to 12 months post-spraying (Fig. 6).

### Insecticide susceptibility status

The exposure of insecticide-susceptible *An. gambiae* s.s. Kisumu strain mosquitoes to filter papers treated with three pyrethroids resulted in 100% mortality, confirming that these insecticide-treated papers were suitable for determining the susceptibility of the wild strain. Control mortality did not exceed 5% in any of the susceptibility bioassays.

**Fig. 7:**
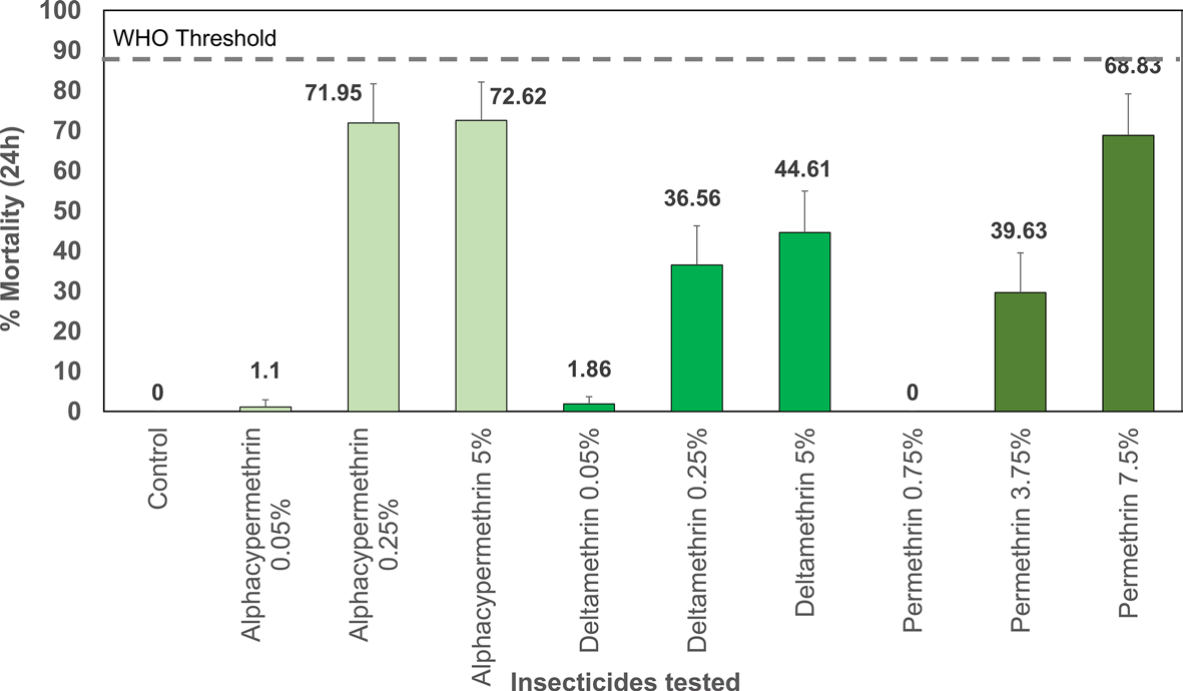
Mortality of wild-population *Anopheles gambiae* s.l. Vallée du Kou mosquitoes exposed to the diagnostic doses and 5 × and 10 × the diagnostic doses of three pyrethroid insecticides in WHO susceptibility and intensity bioassays

WHO tube tests conducted with the wild *An. gambiae* s.l. Vallée du Kou strain demonstrated resistance to all three tested pyrethroid insecticides, with mortality rates < 2% following exposure to diagnostic doses of deltamethrin, permethrin, and alpha-cypermethrin (Fig. 7). Furthermore, the wild *An. gambiae* s.l. Vallée du Kou strain exhibited a high intensity of resistance to alpha-cypermethrin, deltamethrin, and permethrin, with mortality rates < 90% at 10 × discriminating doses (Fig. 7).

**Fig. 8:**
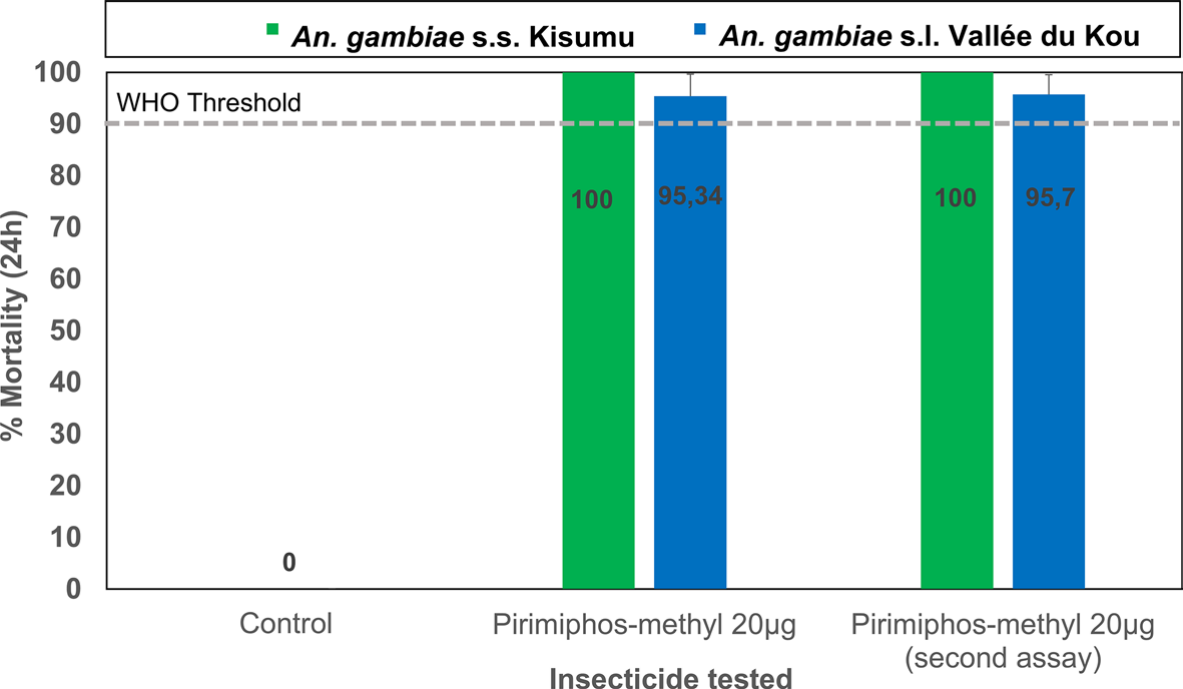
Mortality of susceptible *Anopheles gambiae* s.s. Kisumu strain and wild population *An. gambiae* s.l. Vallée du Kou strain mosquitoes in CDC bottle bioassays treated with a technical grade of pirimiphos-methyl insecticide

In susceptibility assays using 0.25% pirimiphos-methyl in CDC bottle bioassays, the wild *An. gambiae* s.l. Vallée du Kou strain displayed suspected resistance, achieving a mortality rate of 95.34% (CI 95% 90.7–99.3). This finding was confirmed in a repeat bioassay, which yielded a mortality rate of 95.7% (CI 95% 92.2–99.8) (Fig. 8).

**Fig. 9:**
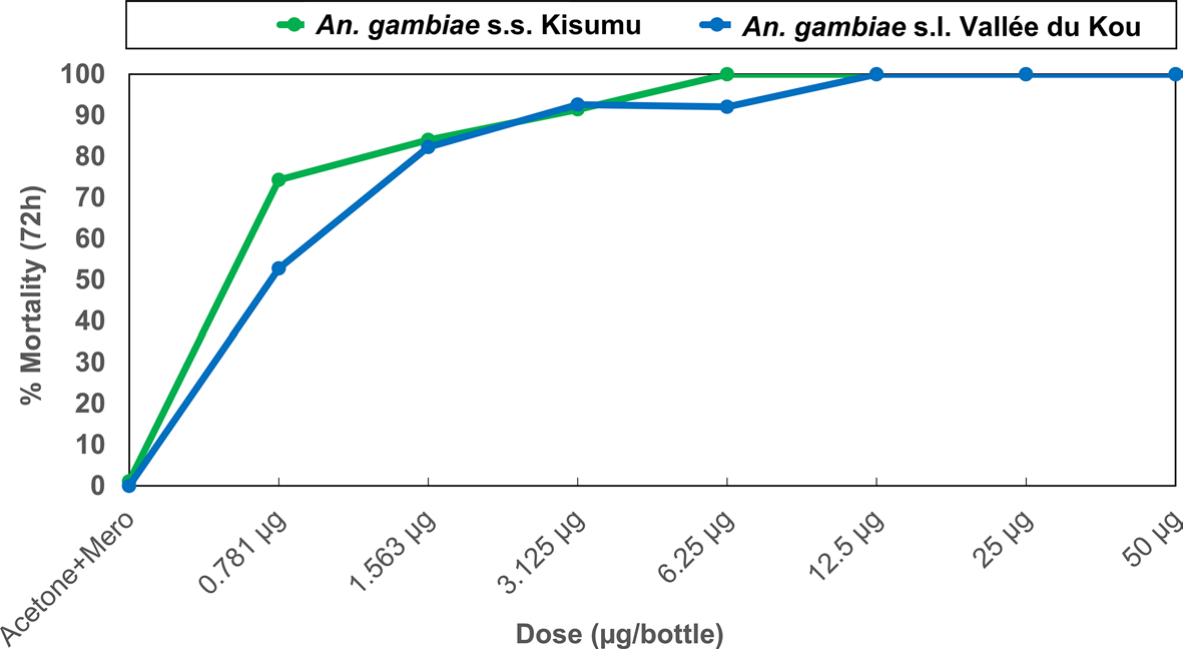
Mortality of susceptible *Anopheles gambiae* s.s. Kisumu and wild *An. gambiae* s.l. Vallée du Kou strain mosquitoes in CDC bottle bioassays treated with different doses of broflanilide insecticide

The results of dose-response assays comparing the mortality rates of the wild *An. gambiae* s.l. Vallée du Kou strain (pyrethroid-resistant) with those of the susceptible *An. gambiae* s.s. Kisumu strain are presented in Fig. 9. Log dosage-probit mortality analysis revealed that the lethal concentration (LC) required to kill 50% (LC50) and 95% (LC95) of the exposed susceptible strain mosquitoes were 0.4 µg/bottle and 3.3 µg/bottle, respectively. For the wild *An. gambiae* s.l. Vallée du Kou strain, the LC50 and LC95 values were 0.6 µg and 4.8 µg, respectively (Table 3). The resistance ratio for the LC50 values was only 1.7, indicating no cross-resistance to broflanilide in the wild strain mosquitoes (Table 3). Moreover, 100% mortality of *An. gambiae* s.s. Kisumu strain mosquitoes was observed at dosages of 6.25 µg/bottle and above, while for the *An. gambiae* s.l. Vallée du Kou strain, 100% mortality occurred at dosages of 12.5 µg/bottle and above (Fig. 9).

**Table 3:** Calculated lethal doses of broflanilide for the insecticide susceptible *Anopheles gambiae* s.s. Kisumu strain and the wild *An. gambiae* s.l. Vallée du Kou strain in CDC bottle bioassays

Strains	Slope (SE)	LC _50_ (95% CI)	LC _95_ (95% CI)	Resistance ratio (95% CI)
Susceptible *An. gambiae* s.s. Kisumu strain	1.7 (0.3)	0.4 (0.2–0.6)	3.3 (2.4–5.3)	–
Wild *An. gambiae* s.l. Vallée du Kou strain	1.9 (0.4)	0.6 (0.3–0.9)	4.8 (3.1–12)	1.7 (1.3–2.4)

### Adverse events and acceptability

Twelve rounds of surveys were conducted in both IRS-sprayed sites, involving 360 respondents (30 individuals per month) at each site. Over the 12 months, 194 males and 166 females were interviewed at the Actellic^®^ 300CS site, while 198 males and 162 females were interviewed at the VECTRON™ T500 site.

Seasonal variation was noted among respondents; during the rainy season (June to October), > 70% of the interviews were conducted with females. In contrast, the dry season saw a higher proportion of males, with > 50% of the interviews being conducted with them. In all houses sprayed with VECTRON™ T500, no adverse events (e.g. sneezing or itching) were reported by selected households or their family members from the 1st month up to 12 months post-spraying (Table 4). At the Actellic^®^ 300CS site, only one inhabitant reported having a cold, and no other adverse events were noted during the rest of the study. All respondents deemed the IRS-based insecticide applications acceptable and expressed a willingness to participate in subsequent rounds of spraying.

**Table 4:** Community’s perceptions of side effects and benefits of IRS with VECTRON^™^ T500 and Actellic® 300CS

Perceived adverse events and benefits of IRS	VECTRON^™^ T500 sprayed site N (%)	Actellic^®^ 300CS sprayed site N (%)
Unpleasant smell	0 (0)	0 (0)
Dizziness	0 (0)	0 (0)
Running nose	0 (0)	0 (0)
Fever	0 (0)	0 (0)
Headache	0 (0)	0 (0)
Cold	0 (0)	1 (0.002)
Sore eyes	0 (0)	0 (0)
Skin irritation	0 (0)	0 (0)
Coughing	0 (0)	0 (0)
Vomiting	0 (0)	0 (0)
Sneezing	0 (0)	0 (0)
Sleeplessness	0 (0)	0 (0)
Was the IRS beneficial?	360 (100)	360 (100)
Did the use of the IRS reduced mosquito bites?	360 (100)	360 (100)
Would you continue IRS intervention?	360 (100)	360 (100)

Thirty people were surveyed each month for 12 months after IRS applications

## Discussion

The primary objective of this study—to evaluate the residual efficacy of VECTRON^™^ T500 compared to Actellic^®^ 300CS against both susceptible and resistant malaria vectors—was successfully achieved. The results clearly demonstrated the comparative performance of both insecticides, providing critical data on their effectiveness. The second objective, which sought to assess the impact of IRS interventions on entomological parameters of malaria transmission in a pyrethroid-resistant, year-round transmission environment, was also fulfilled. The study revealed significant differences between intervention sites and control sites, validating the efficacy of IRS treatments. Moreover, the research was conducted in alignment with WHO recommendations for prospective studies under real-world conditions, confirming that the tested insecticides met WHO specifications for public health use. This adherence to WHO guidelines further solidified the relevance and practical applicability of the findings. The study also addressed the Global Plan for Insecticide Resistance Management (GPIRM) by introducing a new insecticide with a different mode of action, VECTRON™ T500, which serves as an alternative to pyrethroids in areas with significant resistance [37]. Ultimately, the study achieved its overarching goal of providing robust evidence on the effectiveness and community acceptance of IRS insecticides, contributing valuable data to support future malaria control efforts.

VECTRON^™^ T500 is an IRS product that features a novel meta-diamide insecticide, broflanilide, which operates via a distinct mechanism compared to other classes of insecticides currently used in malaria vector control, including pyrethroids. Broflanilide targets a unique binding site on the insect's γ-aminobutyric acid (GABA) receptor. Previous studies have documented that the residual efficacy of VECTRON^™^ T500 resulted in 100% mortality among both susceptible and resistant mosquito strains for a minimum of 6 months [15–17, 21]. These prior findings corroborate those obtained in this community trial. Mortality rates observed in mosquitoes exposed to VECTRON^™^ T500, as demonstrated in cone trials, consistently exceeded the threshold of 80% mortality throughout the 12-month post-application. This extended residual efficacy suggests that VECTRON™ T500 may effectively reduce malaria transmission over a longer duration than IRS formulations of certain other insecticides. Such prolonged residual efficacy would be particularly advantageous in a country like Burkina Faso, where the malaria transmission season spans approximately 4–6 months, ensuring effective control throughout this period. The results of this community trial conclusively indicate that VECTRON^™^ T500 has a more significant impact than Actellic^®^ 300CS on reducing malaria transmission parameters at the community level, particularly when used in high coverage settings alongside pyrethroid-treated insecticide nets (ITNs). Notably, VECTRON^™^ T500 proved to be non-inferior to Actellic^®^ 300CS in decreasing the resting density of free-flying *An. gambiae* s.l. mosquitoes collected indoors and exhibited a significantly higher blood-feeding inhibition than Actellic^®^ 300CS. Moreover, VECTRON^™^ T500 was well accepted by households, with no adverse effects reported. These findings validate the community-level impact of VECTRON^™^ T500 for IRS, demonstrating that its recent inclusion in the WHO list of prequalified products [18] introduces an effective new mode of action for community-based malaria control. Therefore, VECTRON^™^ T500 should be considered a valuable malaria control tool in Burkina Faso, alongside other available vector control strategies such as insecticide-treated bed nets and larval source management.

The emergence of insecticide resistance among malaria vectors against public health insecticides poses a significant threat to achieving malaria elimination goals [38]. As the primary malaria vector control strategy in Burkina Faso relies on universal coverage with ITNs, the documented high intensity of resistance to pyrethroids across the three study sites is particularly concerning. The intensity of pyrethroid resistance serves as a crucial indicator of the potential for diminished efficacy of pyrethroid-based ITNs. The WHO emphasizes that confirmed resistance levels, especially those exceeding ten times the discriminating concentration, highlight the urgent need for the development of effective resistance management strategies [39]. Therefore, it is essential to preserve the efficacy of newly deployed insecticides for malaria vector control. The rotational deployment of IRS products containing insecticides with diverse modes of action can help mitigate the selection pressure for the proliferation of insecticide resistance genes within malaria vector populations and is thus recommended for the proactive management of insecticide resistance [37]. However, this strategy has yet to be adequately explored for malaria vector control, owing to the historically limited selection of insecticide modes of action available for IRS [40]. The susceptibility of the wild mosquito vectors to broflanilide, as demonstrated in CDC bottle bioassays, revealed no cross-resistance through mechanisms linked to other insecticides present in this population. In contrast, the local vector population displayed suspected resistance to pirimiphos-methyl, the insecticide utilized in the Actellic^®^ 300CS formulation serving as the reference product in this study.

## Conclusions

VECTRON™ T500 demonstrated non-inferiority to Actellic^®^ 300CS in reducing malaria transmission parameters from pyrethroid-resistant vectors when applied as insecticide residual spraying (IRS) in a community trial in Burkina Faso. The insecticide exhibited prolonged efficacy on all types of walls for 12 months post-application, effectively covering the malaria transmission period in the country. Additionally, VECTRON^™^ T500 received high acceptance from community members at the sprayed sites. Overall, VECTRON^™^ T500 represents a promising solution for the control of pyrethroid-resistant vectors across various contexts.

## Data Availability

No datasets were generated or analyzed during the current study.

## Abbreviations

CI95%: Confidence interval at 95%
GPIRM: Global Plan for Insecticide Resistance Management
IRAC: Insecticide Resistance Action Committee
IRD: Indoor resting density
IRS: Indoor residual praying
ITNs: Insecticide-treated nets
IVCC: Innovative Vector Control Consortium
LC: Lethal concentration
LSHTM: London School for Health and Tropical Medicine
RR: Relative ratio
VK: Vallée du Kou
WHO: World Health Organization
